# The applied study to improve the treatment of knee sports injuries in ultimate frisbee players based on personalized exercise prescription: a randomized controlled trial

**DOI:** 10.3389/fpubh.2024.1441790

**Published:** 2024-09-17

**Authors:** Shangmin Chen, Lin Du, Yongshan Gao, Haorui Li, Yanxun Zheng, Lei Xie, Zhigang Zhong

**Affiliations:** ^1^Sports Medicine Center, The First Affiliated Hospital of Shantou University Medical College, Shantou, China; ^2^Sports Medicine Institute, Shantou University Medical College, Shantou, China; ^3^School of Public Health, Shantou University, Shantou, China; ^4^Injury Prevention Research Center, Shantou University Medical College, Shantou, China

**Keywords:** sports injury, sports prescription, ultimate frisbee, knee, risk factors

## Abstract

**Objective:**

Ultimate frisbee can lead to severe sports injuries, especially joint injuries in the lower limbs, such as knee meniscus injuries. This study examines the impact of personalized exercise therapy on knee meniscus injuries in ultimate frisbee players in the Lingnan region of China.

**Methods:**

Seventy-six patients with confirmed meniscal injuries participated in the study, divided into an intervention group (*n* = 38) and a control group (*n* = 38). The control group received standard treatment, including drug therapy and physical therapy. The intervention group received standard treatment plus a personalized exercise regimen based on FITT-VP (frequency, intensity, time, type, volume, and progression) principles, incorporating strength training, aerobic exercise, flexibility training, neuromuscular training, and aquatic exercise. This program was monitored and adjusted over a six-month period through both online and offline methods. The primary outcomes were joint range of motion (ROM), thigh circumference atrophy index (TCAI), Lysholm Rating Scale (LRS) scores, and visual analog scores (VAS). The secondary outcome was the International Knee Documentation Committee (IKDC) score. Data were collected before the intervention, and at 1 month and 6 months after the intervention. Statistical analysis was conducted using SPSS 24.0 and GraphPad 10.0, with a significance level set at *α* = 0.05.

**Results:**

After 1 month, the intervention group showed significantly better results in ROM (116.67 ± 9.063), LRS score (86.316 ± 3.750), and IKDC score (80.473 ± 5.421) compared to the control group (111.784 ± 4.778, 82.579 ± 3.818, and 77.684 ± 4.430, respectively) (*p* < 0.05). The TCAI (3.219 ± 1.889) and VAS score (1.921 ± 0.673) in the intervention group were significantly lower than those in the control group (5.228 ± 2.131 and 2.710 ± 1.112, respectively) (*p* < 0.01). After 6 months, the differences in LRS and VAS scores between the groups were not significant. However, the intervention group continued to show significant improvements in ROM (134.934 ± 3.011), TCAI (1.107 ± 1.158), and IKDC score (93.315 ± 1.847) compared to the control group (125.395 ± 18.554, 4.574 ± 1.109, and 87.789 ± 4.437, respectively) (*p* < 0.05).

**Conclusion:**

Personalized exercise prescriptions offer significant therapeutic and rehabilitative benefits for ultimate frisbee players with knee meniscus injuries. This approach helps to reduce symptoms, alleviate pain, and improve joint function, muscle strength, and athletic performance after sports-related injuries.

## 1 Introduction

With the increasing popularity of Frisbees, sports injuries has come to the forefront. The ultimate Frisbee is a noncontact or limited-contact sport involving a full range of movements, such as endurance sprinting, cutting, spinning, and jumping, which could result in sports injuries of different severity among participants of different age groups and sports levels. The incidence rate of sports injuries caused by ultimate Frisbee is between 48.0 and 68.0% (1–3). In a Hong Kong survey, it was found that the incidence of Frisbee injuries among Hong Kong athletes was 5–6 times lower than that in the United States (4). The incidence rate of the knee injuries for frisbee players is 14%. In professional leagues, 17.5% of frisbee players are injured due to an incorrect landing position, with the most common site of injury being the knee (1).

In ultimate frisbee, meniscal injuries are prevalent knee injuries that can impact performance and lead to long-term impairments and reduced quality of life (5). Despite a high incidence rate among athletes, exact numbers are elusive due to insufficient systematic research. Although traditional treatments such as surgery and medication are widely used in clinical practice (6), people also notice that exercise can help prevent osteoporosis, accelerate healing after fracture, and significantly aid in body function recovery (7–14). Exercise is recognized for its benefits in preventing osteoporosis, hastening fracture recovery, and aiding overall body function (15–17). Tailored exercise programs, including isometric strength training and aerobic exercise, have been shown to enhance joint stability, alleviate pain, and improve the knee’s dynamic balance, which is vital for the recovery of meniscal injury (14, 18–20). Moreover, regular exercise interventions have a positive impact on patients’ quality of life and mobility, with benefits confirmed through long-term follow-up studies (18, 21).

The concept of “Personal exercise prescription” involves tailoring exercise regimens to individual needs, considering six factors (FITT-VP): exercise frequency (F), exercise intensity (I), exercise time (T), exercise type (T), exercise volume (V), and exercise progression (P) (22). This personalized approach has shown potential in improving musculoskeletal health, which is the focus of our investigation (23, 24). Although some existing studies have focused on the impact of exercise therapy on meniscal injuries (16, 25, 26), there is a significant gap between the research and the application of personalized exercise prescriptions in this field (27). Current research tends to concentrate on short-term rehabilitation outcomes (19, 28), while there is a relative lack of studies focusing on the long-term effects of exercise therapy, improvements in quality of life, and prevention of re-injury. The importance of exercise therapy in the rehabilitation of meniscal injuries has been acknowledged in some studies. However, the literature predominantly emphasizes surgical and pharmacological treatments. To bridge the gap, our research aims to evaluate the effectiveness of personalized exercise therapy for meniscal injury rehabilitation, offering more comprehensive guidance and support on the application of exercise prescriptions in chronic musculoskeletal diseases, as well as further exploring their benefits in the diagnosis and treatment of sports injuries. The anticipated outcomes will provide a scientific foundation for the standard treatment of meniscal injuries, promote the development of personalized treatment strategies, and significantly contribute to improving patients’ quality of life and reducing the societal burden, while also providing theoretical support for the application of exercise prescriptions in sports rehabilitation.

## 2 Methods

### 2.1 Study design and participants

This study was designed as a prospective randomized controlled trial (RCT) to evaluate the effects of personalized exercise in the recovery of meniscal knee injuries of frisbee players. This study focused on patients who had knee meniscus injuries due to ultimate frisbee sports within the last 6 months before enrollment. These patients participated in the Cross-sectional Survey of Ultimate Frisbee Sports Injuries from January 2022 to January 2023. The patients were treated at a hospital located in the eastern region of Guangdong Province in China from July 2022 to December 2023. The Department of Sports Medicine in this hospital is a locally renowned sports medicine center and serves as the medical support unit for frisbee sports clubs in the region.

The inclusion criteria of the study were participation in ultimate frisbee sports for more than 6 months, 18–45 years old, adequate hearing and vision, clear consciousness, good verbal communication skills, voluntary participation in the survey, The patients has been diagnosed by a doctor with symptoms of meniscus injury and confirmed by medical imaging (I-II degree). Types of meniscal injuries included in this study were tears, degeneration, and cysts.

The exclusion criteria included meniscus injury of degree III or those combined with other knee diseases, lower limb surgery within 6 months, cognitive impairment, severe hearing or vision impairment, severe heart, brain, kidney or other organ dysfunction, limb hemiplegia, non-cooperation with the intervention experiment, and being lost to visit.

**Figure 1:**
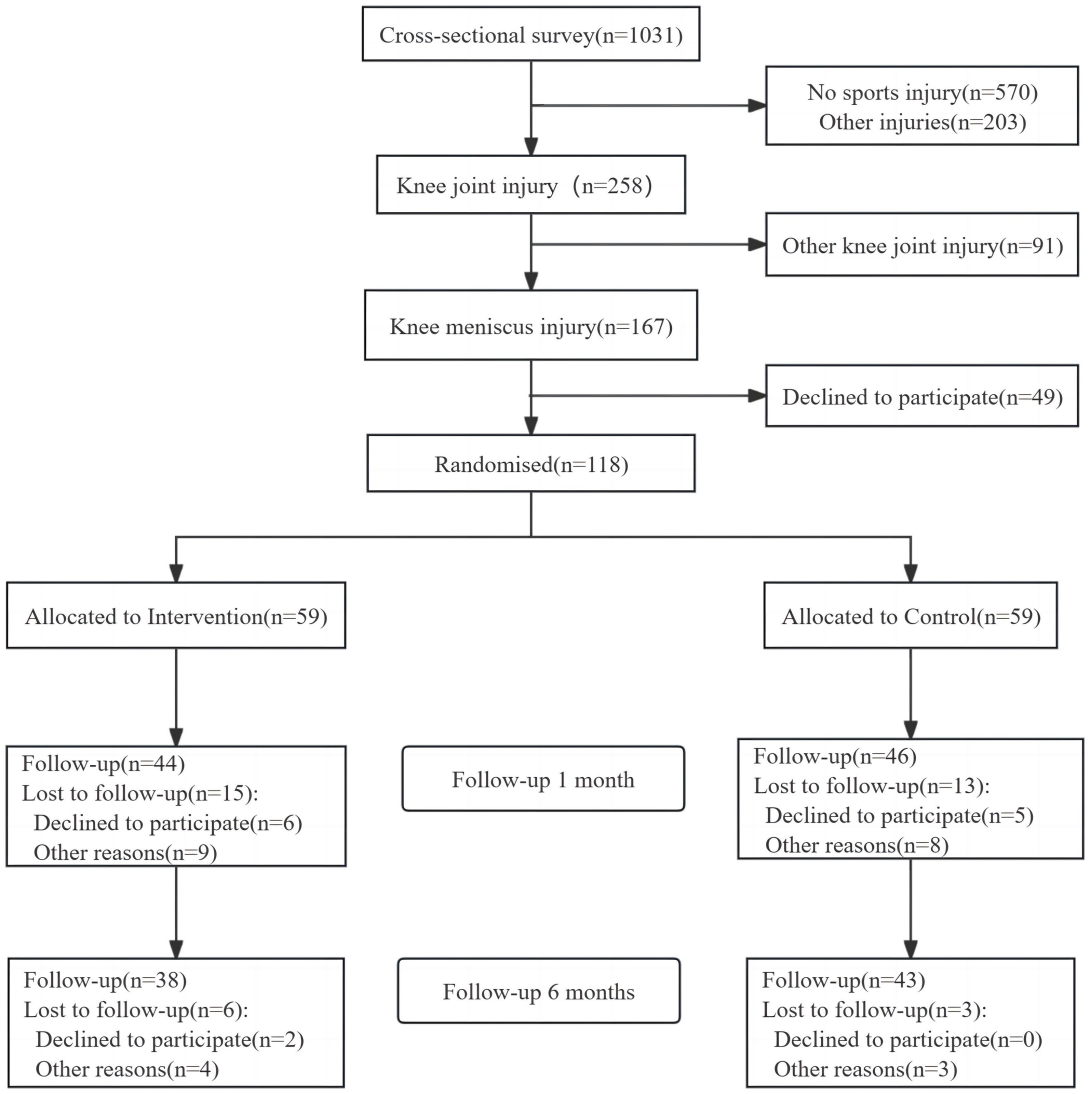
Study participant flowchart (CON, control group; EPI, exercise prescription intervention group).

One hundred and eighteen participants randomly assigned them to either the intervention group (59 participants) or the control group (59 participants) in a 1:1 ratio. The randomization process was conducted using a computer-generated sequence to ensure allocation concealment. After 6 months interventions, excluding those due to various reasons had quitted the research and we have to follow the 1:1 matching requirement. There are 76 participants include in this research (38 participants in each group) (see Figure 1).

All participants signed an informed consent form before enrollment. A member of the investigator’s team evaluated disease-related information about the patients via a face-to-face meeting. The data collection form was completed and double-checked by the other team members to ensure the accuracy and completeness of the collected data. The study received approval from The First Affiliated Hospital of Shantou University Medical College Ethics Committee (B-2023-107), and all participants provided written informed consent. The study followed the principles of the Declaration of Helsinki to ensure participant privacy and data security.

### 2.2 Intervention procedures

The control group (CON group): Received a standard treatment, including conventional conservative therapy (such as braking and resting) and common interventions (e.g., providing science articles and video push information related to meniscus injury). No exercise prescription was provided to the control group.

Exercise prescription intervention group (EPI group): In addition to standard care, participates received a personalized exercise prescription based on the FITT-VP principle. The exercise prescription intervention included online intervention (online push exercise prescription, related sports injury popular science articles, videos, etc.) as well as offline intervention (offline assessment and individualized adjustment of exercise prescription in the first, second, third and sixth months). Through phone calls and microletter platforms, we monitored the pain condition and the patients’ ability to tolerate the exercise prescription until the end of the intervention in the intervention group. Meanwhile, questionnaires, including the Lysholm Rating Scale, VAS pain scale, and IKDC score, were administered before the intervention and in in the first and sixth months after the start of the intervention. Clinical indicators, including joint mobility ROM, thigh circumference atrophy index, were measured.

#### 2.2.1 Exercise prescription formulation

The exercise prescription formulators in this study received and completed professional exercise prescription training and obtained clinical exercise prescriber certification. When formulated, the intervention group’s exercise habits were combined to determine whether there was a risk of injury, assess exercise goals and develop a plan in phases, in order to ensure the implementation of exercise prescription, provide follow-up feedback and assess and compare to prevent possible exercise risks [21]. The stages of exercise prescription intervention in this study were divided into preintervention (after injury) and mid- to late (1 month) intervention, preintervention to relieve knee pain and restore essential knee function for the primary purpose of sports enthusiasts, and mid- to late intervention to accelerate the recovery of knee function for sports enthusiasts, strengthen the muscles around the knee joint, and improve cardiorespiratory fitness and athleticism for the primary purpose. Currently, commonly used exercise modalities include strength training, aerobic training, flexibility training, neuromuscular training and aquatic exercise. Under the FITT-VP principle, the exercise intensity was gradually adjusted during training. The preintervention data are shown in Supplementary Table 1, and the mid- and postintervention data are shown in Supplementary Table 2.

### 2.3 Outcome measures

Both primary and secondary outcome indicators were assessed before the start of the intervention (baseline), 1 month after the intervention, and 6 months after the intervention. When clinical indicators were measured, subjects were allowed to perform a 5-min warm-up of ROM self-stretching within a pain-free range. Physicians measured and recorded differences in thigh circumference and joint mobility.

#### 2.3.1 Primary outcomes

The primary outcome indicators used to assess the degree of pain were the joint range of motion (ROM), the thigh circumference atrophy index (TCAI), the Lysholm Rating Scale score (LRS), and the visual analog score (VAS).

Maximum flexion range of motion (ROM) of the knee: the angle at which the joint flexes within the pain-free range, assessing the severity of knee injury or disease. The ROM was measured with a protractor. MCID: 10° improvement.

Thigh circumference atrophy index (TCAI): the thigh circumference was calculated by defining 10 cm above the patella bilaterally and then dividing the difference between the thigh circumference on the healthy and affected sides by the thigh circumference on the healthy side. A greater atrophy index indicates a more significant difference in quadriceps circumference between the affected and healthy sides, indicating a greater degree of atrophy of the quadriceps muscle on the affected side (29). MCID: 10% reduction.

Lysholm Rating Scale (LRS): the Lysholm Rating Scale is a commonly used tool for assessing knee function and symptoms, especially when evaluating functional recovery after knee injury (30). This rating scale consists of eight items, including walking limitations, gait, support, going up and down stairs, joint mobility, swelling, sensation, and stability. The total score ranges from 0 to 100, with higher scores representing better knee function and less severe symptoms. MCID: 10-point improvement.

Visual Analog Scale (VAS): the VAS pain scale assesses the intensity of pain, ranging from 0 (no pain) to 10 or 100 (worst pain). Higher scores indicate more severe pain, and further away from the “no pain” end of the scale, the subject is labeled. It is widely used to assess various types of pain, including acute pain, chronic pain, and postsurgical pain, which helps determine treatment effects, monitor changes in pain, and guide clinical decision-making. MCID: 2-point reduction.

#### 2.3.2 Secondary outcomes

The secondary outcome indicators was the International Knee Documentation Committee (IKDC) score.

The IKDC scale International Knee Documentation Committee score consists of 10 items on knee function and eight items on knee ligament examination, including joint pain, level of motion and ability to perform daily activities, with a total score of 0–100 (31). Higher scores indicate better joint function. The IKDC scale can reliably, validly, and sensitively assess changes in a patient’s joint function (32). The IKDC questionnaire is frequently used in knee studies and also easier to complete and understand than other questionnaires (33, 34). MCID: 10-point improvement.

### 2.4 Sample size estimation and statistical analysis

Regarding ROM as the primary outcome, the standardized ROM value at 1 month after intervention was 120°. Previous studies mostly grouped knee joints at intervals of 10° when assessing knee joint functional status. Therefore, our study assumed a difference of 10° between the two groups at 1 week, with the EPI group at 125° and the CON group at 115° (31). The standard deviation for both groups was set at 10°, with a significance level *α* of 0.05 and 1-*β* of 0.90. This study was a randomized controlled trial (RCT) with a 1:1 ratio of participants between the two groups. Calculations were performed using PASS software. The sample size for each group was calculated to be 23. Considering a dropout rate of approximately 20% at the second follow-up, the total sample size was set at 56.This study included 76 patients after 6 months intervention, which is more than the required sample size.

Epidata 3.1 software was used to establish the database and enter the data. To support the replicability of this study, all data were recorded using a standardized electronic data collection system and double-input verification was used to ensure data accuracy. The statistical analysis plan was formulated before the study began and adhered to strictly throughout the research process. SPSS 24.0 and GraphPad Prism 10.0 software were used for the statistical analysis of the data. The independent sample t-test was employed to compare the means of continuous variables between the two groups, while the chi-square test was utilized for categorical variables. Additionally, analysis of variance (ANOVA) was conducted to investigate the differences in all study variables across the cohorts. The assessment indicators of the follow-up and the measurement information that satisfied the normal distribution were expressed as (x ± s). The two samples were compared using an independent samples t test, and the significance level of all the statistical tests was set to be *α* = 0.05.

## 3 Results

Seventy-six study participants were enrolled in the study and randomized controlled study and were matched 1:1 according to sex and exercise level status in the EPI group and the CON group. The flow of participant inclusion through the study is shown in Figure 1. Table 1 presents the baseline characteristics demonstrating no significant differences between groups in terms of age, weight, height, BMI, initial ROM, LRS, IKDC score, VAS score, and TCAI (all *p* < 0.05).

**Table 1:** Mean (SD) baseline characteristics of the participants (*n* = 76, x ± s).

Characteristics	CON (*n* = 38)	EPI (*n* = 38)	*p* value
Age	26.26 ± 4.35	25.84 ± 4.51	0.678
Weight (kg)	21.97 ± 3.02	21.15 ± 2.213	0.186
Height (cm)	74.895 ± 4.151	75.474 ± 3.493	0.513
ROM	97.816 ± 8.868	97.079 ± 10.070	0.736
IKDC	71.079 ± 3.996	70.131 ± 3.677	0.286
VAS	3.763 ± 1.195	3.711 ± 0.802	0.822
TCAI	0.062 ± 0.019	0.060 ± 0.024	0.640
BMI	21.97 ± 3.02	21.15 ± 2.213	0.186
Lysholm	74.895 ± 4.151	75.474 ± 3.493	0.513

CON, control group; EPI, exercise prescription intervention group; BMI, body mass index; ROM, various indicators such as range of motion; IKDC, International Knee Documentation Committee; VAS, visual analog scale; TCAI, thigh circumference atrophy index.

### 3.1 ROM

**Figure 2:**
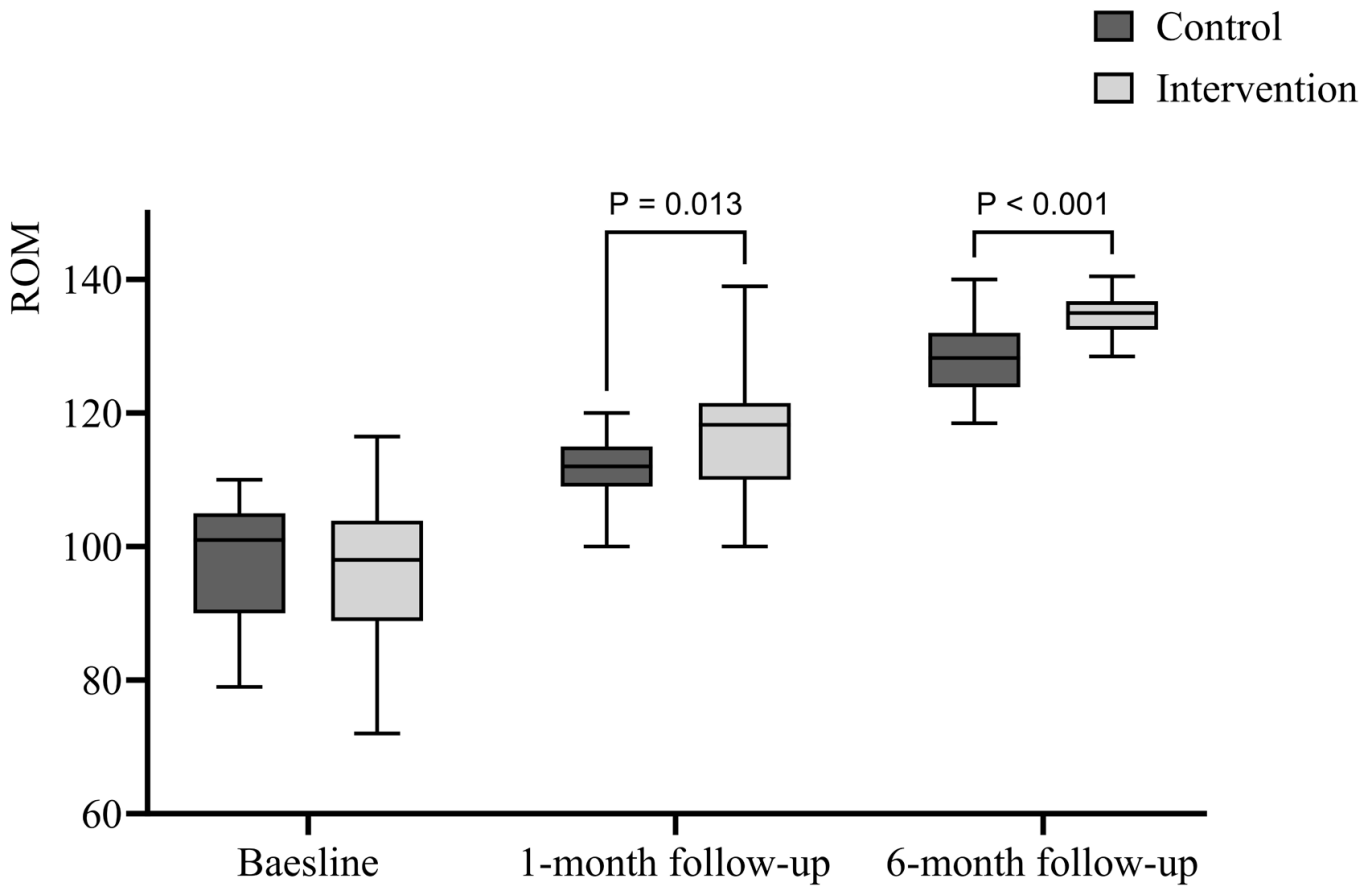
Box plot of the ROM.

After 1 month of follow-up, the ROM in the EPI group (116.67 ± 9.063) was significantly greater than that in the CON group (111.784 ± 4.778) (*p* = 0.004). This improvement was maintained at 6 months, with the EPI group achieving a ROM of (134.934 ± 3.011), significantly greater than the CON group (128.290 ± 5.748) (*p* < 0.001). Figure 2 shows that the improvement in ROM in the EPI group became more obvious and the difference of ROM between the intervention group and the control group was significantly after one and 6 months of follow-up.

### 3.2 TCAI

**Figure 3:**
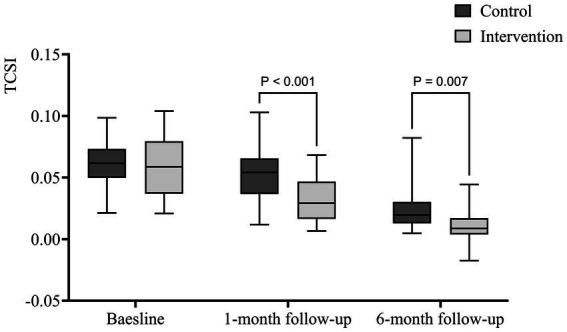
Box plot of the TCAI.

The EPI group exhibited a significantly lower TCAI at 1 month (0.032 ± 0.019) compared to the CON group (0.0523 ± 0.021) (*p* < 0.001). This difference persisted at 6 months, with the EPI group’s TCAI at (0.011 ± 0.012), significantly lower than the CON group’s (0.024 ± 0.017) (*p* = 0.007). Figure 3 shows that the muscles improved and the different of TCAI between two groups was significant after 1 month and 6 months of follow-up.

### 3.3 Lysholm Rating Scale

**Figure 4:**
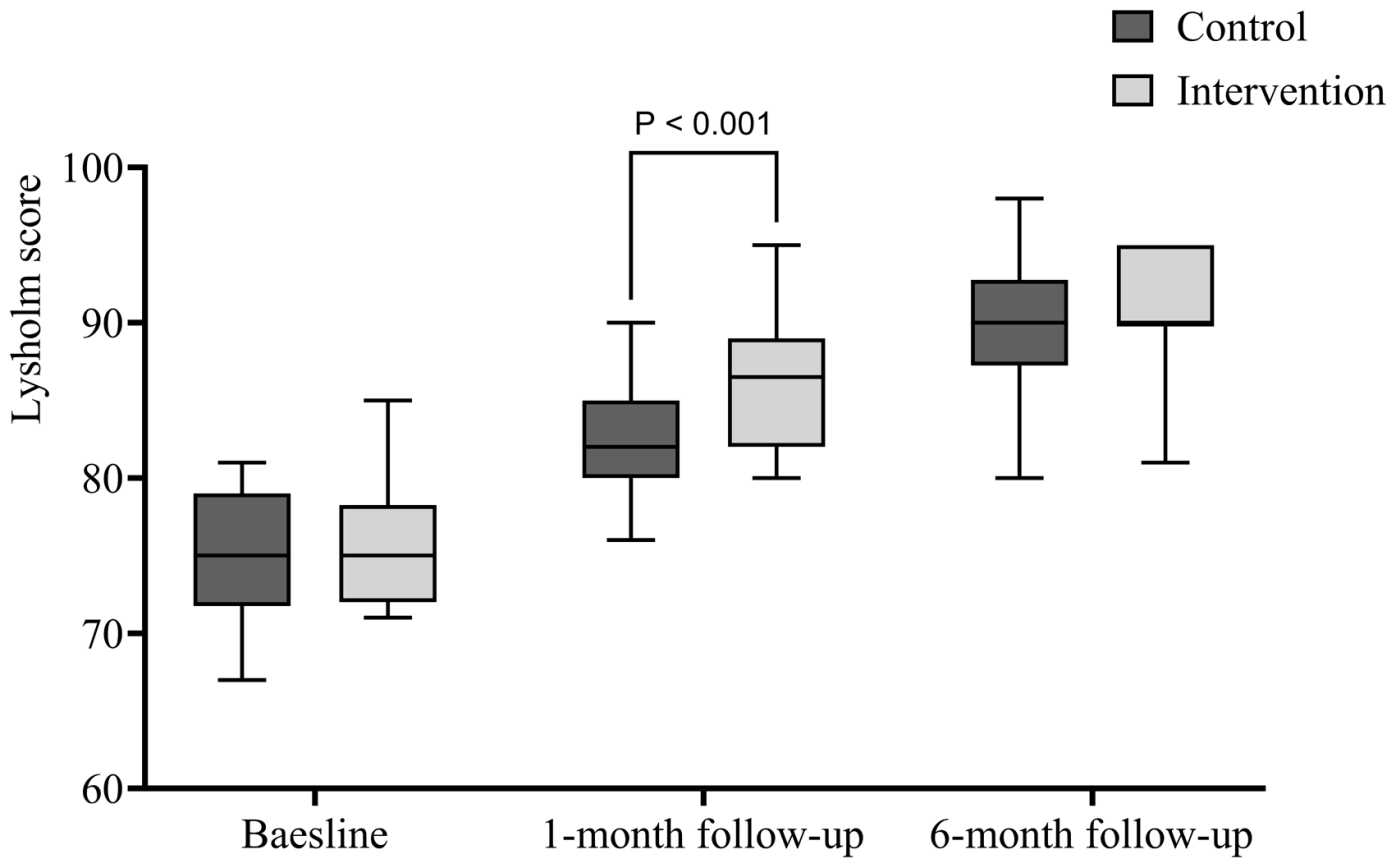
Box plot of the Lysholm Rating Scale score.

The EPI group’s LRS score at 1 month (86.316 ± 3.750) was significantly higher than the CON group’s (82.579 ± 3.818) (*p* < 0.001). However, by 6 months, the difference was no longer significant. As shown in Figure 4, the difference in the Lysholm functional score between the EPI group and the CON group was statistically significant after 1 month of intervention (*p* < 0.001); there was no significant difference between the two groups after 6 months of follow-up.

### 3.4 VAS scale

**Figure 5:**
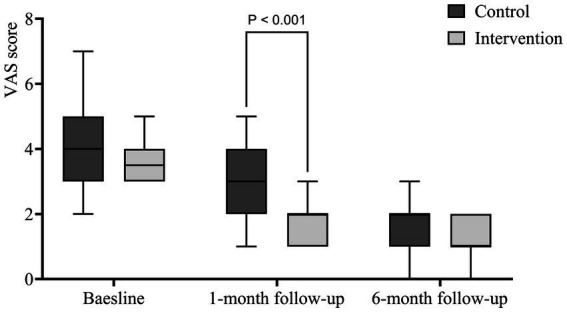
Box plot of the VAS score.

The EPI group had a significantly lower VAS score at 1 month (1.921 ± 0.673) than the CON group (2.710 ± 1.112) (*p* < 0.001). The difference was not significant at 6 months. Figure 5 shows no statistically significant difference between the two groups before and after 6 months of intervention but the improvement of VAS pain score was significant in the EPI group compared with the CON group after 1 month of follow-up.

### 3.5 IKDC score scale

The IKDC score in the EPI group (80.473 ± 5.421) was significantly greater (*p* < 0.05) than that in the CON group (77.684 ± 4.430) after 1 month. This trend continued at 6 months, with the EPI group scoring 93.315 ± 1.847, significantly higher than the CON group’s 87.789 ± 4.437 (*p* < 0.001), see Table 2 for details.

**Table 2:** IKDC scores at the 1- and 6-month follow-ups (mean ± SD).

IKDC	CON	EPI	*p*
1-month follow-up	77.684 ± 4.430	80.473 ± 5.421	0.016
6-month follow-up	87.789 ± 4.437	93.315 ± 1.847	<0.001
1-month follow-up-6-month follow-up	10.105 ± 3.608	12.842 ± 5.664	0.005

## 4 Discussion

In our recent epidemiological study of ultimate frisbee athletes in southern China, we found a high incidence of sports injuries, with 464 out of 1,031 participants (45.0%) reporting injuries over the past year, predominantly in the lower extremities. The most frequent injury sites were the knee (25.5%), thigh (20.5%), and ankle (12.2%), with sprains and ligament injuries making up 35.6% of these cases. These findings highlight the significant risk factors associated with ultimate frisbee, such as high intensity, rapid directional changes, and jumps—particularly detrimental to the knee. This background informs the current study’s focus on evaluating personalized exercise prescriptions to mitigate knee injuries among ultimate frisbee players. The exercise prescriptions used in this study were based on the 2023 Chinese Expert Consensus on Exercise Prescription (22). The early, mid- and long-term exercise prescriptions were formulated according to the FITT-VP principle and combined with the characteristics of Ultimate Frisbee sports, the characteristics of injuries in Frisbee sports enthusiasts, and their exercise preferences. Ultimate Frisbee sports enthusiasts usually do not have underlying diseases or other problems in the standard body systems. The risk of various types of accidents during exercise is small. Only basic assessment and evaluation of effects after exercise, such as heart rate, pulse and body weight, are needed to ensure that the primary indicators are averaged.

This study aimed to assess the effectiveness of targeted exercise in treating knee meniscus injuries in Ultimate Frisbee sports enthusiasts. Based on our results, within 1 month after the early intervention, the VAS score (1.921 ± 0.673 vs. 2.710 ± 1.112), Lysholm score (86.316 ± 3.750 vs. 82.579 ± 3.818), TCAI (0.032 ± 0.019 vs. 0.0523 ± 0.021) and ROM (116.67 ± 9.063 vs. 111.784 ± 4.778) of the ultimate Frisbee sports enthusiasts who received the exercise intervention improved more significantly than those of the control group. It provided the evidence that the short-term exercise prescription could effectively alleviate the pain sensation of the injured and promote the rehabilitation of joint function to restore a certain degree of quality of life, which could enhance the adaptability of patients to sports and alleviate the pain caused by injuries. These results are consistent with the literature showing that exercise prescription is efficacious in improving joint function, reducing pain and improving overall athletic performance in the short term (35, 36). This positive effect was partially maintained in the long term (6 months postintervention), consistent with the findings of previous studies concerning the effects of exercise interventions on sports injury recovery (37, 38). Assessment of the outcome indicators at 6 months postintervention revealed significant differences in IKDC scores (93.315 ± 1.847 vs. 87.789 ± 4.437), ROM (134.934 ± 3.011 vs. 125.395 ± 18.554), and TCAI (0.011 ± 0.012 vs. 0.024 ± 0.017). The exercise prescription group showed more pronounced improvements of these three indicators than the control group, revealing better muscle strength, better recovery of joint function, and a greater quality of life in the patients who received the exercise prescription intervention. This also confirmed the effectiveness and continuity of exercise prescription interventions for sports injuries. These short-term improvements may be mainly due to the targeted plyometric and functional exercises included in the exercise prescription, which enhance muscle strength, increase joint stability, and improve the mechanical environment of the joints, thus reducing the abnormal stress distribution in the joints and decreasing the pain caused by instability.

Studies have shown that ROM is closely related to the demands of daily activities. Patients can meet minimum standards of daily activities when mobility is ≥90° (ROM) and can squat easily when mobility is >120° (28). The sooner patients are helped to achieve greater ROM, the more patients will experience improved quality of life and satisfaction (37, 38). In this study, the ROM was slightly greater in the EPI group than in the CON group at the one-month follow-up after the intervention, similar to the findings from other studies of the knee (39, 40). By 6 months, the EPI group showed a more significant improvement in ROM than the CON group. This was mainly because patients were trained with basic training 1 month after the intervention, after which the content increased, and patients in the EPI group could receive more excitement and varied one-on-one instructions. In addition, patients’ attention to rehabilitation training tended to decrease over time, and psychological barriers gradually increased, as shown in previous studies (31, 41). At this time, timely health education and exercise prescription can motivate patients to maintain a high level of adherence to achieve better rehabilitation outcomes.

The study showed both groups have substantial muscle improvement after 1 and 6 months of follow-up, and the degree of atrophy of the quadriceps femoris (QF) muscle is considered to be a critical factor in knee malfunction (42). The activity of the QF muscle decreased due to problems such as limited knee flexion after injury. At the same time, skeletal muscle and ligament injuries result in inadequate oxygen supply and adjacent capillary damage, which leads to QF atrophy (43, 44). In the present study, patients who underwent EPI received frequent training reminders as well, which led to better performance reduction in QF atrophy. A reduction in QF atrophy accompanied by more significant pain relief may contribute to a more remarkable improvement in ROM (31).

The success of exercise prescription relies heavily on long-term patient compliance. However, these positive results showed some attenuation at long-term follow-up, especially for the Lysholm score. The lack of long-term effects may reduce the overall success of the treatment, which may indicate that the long-term effects of exercise need to be maintained through consistent and regular exercise modifications. There are several reasons for these diminished long-term effects. First, patients may need ongoing supervision to maintain the prescribed frequency and intensity of exercise. Second, the patient’s understanding of the exercise prescription may need to be revised, avoiding improper implementation or lack of effectiveness.

Flexion pain, a significant factor in patient dissatisfaction, often discourages ongoing participation in therapeutic programs when immediate results are not evident (45). Our findings confirm that standardized training, which has been shown to alleviate such pain by improving medial femoral trajectory and correcting patellofemoral joint abnormalities, is crucial in enhancing patient outcomes. Unlike general reviews that outline potential benefits broadly, our study specifically observed reductions in patellofemoral pain syndrome, aligning with prior research (31, 46). Furthermore, our research addresses a common challenge in sports medicine: the interruption of prescribed exercises due to fear of re-injury and pain (31). Effective communication and a well-shared rehabilitation program can significantly alter patients’ perception of training intensity, mitigating fear-avoidance behaviors and improving adherence to prescribed regimens. Our study recommends regular assessments and adaptable adjustments to exercise prescriptions based on patient feedback to enhance long-term adherence, which is essential for sustaining the benefits of exercise interventions. Our results support this approach, suggesting that patient education and the use of technological tools like wearable devices and mobile health apps are effective in providing real-time feedback and promoting engagement. Moreover, the psychological support component of our interventions, which provided therapeutic support for coping with motivational declines, pain management, and mood swings during recovery, proved vital. Based on our observations, incorporating adherence monitoring into exercise prescription programs, possibly through community support or online supervision, appears promising. Thus, while earlier literature provides a foundation, our study contributes to a more nuanced understanding of how specific interventions can be optimized to improve recovery outcomes in patients with sports-related injuries.

Based on our study, the clinical implications of the personalized exercise prescription in managing knee meniscus injuries in lower extremity sports could be summarized as following. Improved Rehabilitation Outcomes, Exercise prescription interventions enhance athletes’ muscle strength, proprioception, joint mobility, and activity levels, resulting in faster recovery and greater functional benefits compared to traditional rehabilitation methods (14, 47, 48). To integrate personalized exercise prescriptions into traditional treatment methods, such as physical therapy and medication, could formulate a comprehensive rehabilitation program, which could foster quicker recovery and long-term joint health. Functional Improvement, Customized exercise regimens lead to greater functional improvement in knee stability and mobility. Besides, the implementation of preventive exercise interventions in the early stages of Frisbee sports, which not only helps reduce the incidence of initial injuries but also accelerates recovery and lowers recurrence rates of post-injury. Adherence and Motivation, Personalized exercise plans enhance patient engagement and long-term adherence through regular adjustments and modern technology monitoring (49–51). Clinicians should also focus on educating patients about exercise prescriptions and providing psychological support during treatment.

Our study has certainly some limitations. First, the small sample size may limit the broad applicability of the statistical results. Due to the effects of individual differences, varying amounts of exercise, etc., a larger intervention sample size is needed for the analysis of postintervention outcome indicators. Second, the study lacked of long-term follow-up data, which made it difficult to assess the impact of exercise prescription on the long-term health status of patients. Convergent exercise prescriptions for different individuals during the intervention implementation phase may fail to maximize recovery. Furthermore, targeted research is needed to determine the effectiveness of exercise prescriptions for sports injuries. Additionally, the failure to comprehensively monitor the quality of exercise execution in all patients may have influenced the assessment of intervention effectiveness. Based on our results, future research should consider expanding the sample size and designing a long-term follow-up mechanism to assess the sustained effects of exercise prescription and long-term patient rehabilitation. Meanwhile, studies should integrate new technologies, such as wearable devices, to monitor patients’ activities and ensure that exercise prescriptions are performed with quality and adjusted in real time.

## 5 Conclusion

Exercise prescription has positive therapeutic and rehabilitative effects on frisbee sports enthusiasts with knee meniscus injuries. These interventions can reduce postinjury pain in sports enthusiasts, improve joint function, and increase muscle levels. These findings can provide a reference for clinicians to better diagnose and treat exercise-related diseases.

## Data Availability

The original contributions presented in the study are included in the article/Supplementary material, further inquiries can be directed to the corresponding author.
