# Supplementary material for: The applied study to improve the treatment of knee sports injuries in ultimate frisbee players based on personalized exercise prescription: a randomized controlled trial
Source: Front Public Health. 2024 Sep 17;12:1441790. doi: 10.3389/fpubh.2024.1441790 (PMC11442233; doi:10.3389/fpubh.2024.1441790)
Supplement: Supplementary file 1 [file Table_1.DOCX]

Supplementary Material

# Supplementary Tables

Supplementary Table 1: Prescription for Meniscus Injury in Ultimate Frisbee Enthusiasts (0-1 month)

| **Exercise Forms** | **Aerobic Exercise** | **Resistance Training** | **Weight Training** |
| --- | --- | --- | --- |
| **Types of Exercise** | □Quadriceps Isometric Contraction  □Hamstring Isometric Contraction  □Ankle Pumps  □Straight Leg Raises Training  □Heel Slides | □Resistance Band Heel Slide Training  □Weighted Straight Leg Raises Training | □Bodyweight Squats Training  □Gait Balance Exercises  □Corrective Gait Training |
| **Intensity/Frequency** | 50 repetitions each time, 3 sets per day; light to moderate, ≥3 times per week | The weight should be such that there is no knee joint soreness the next day | Squats: 3 sets of 10 repetitions each time; gait exercises for 10 minutes. At least 3 times per week. |
| **Time** | Intervention starts at baseline -1 week | Intervention weeks 1-4 | Intervention weeks 2-4 |
| **Weekly Exercise Volume** | One cycle per day | One cycle per day | One cycle per day |
| **Exercise Progression** | None | None | None |
| **Precautions** | 1. Quadriceps Isometric Contraction Exercise: Tighten and relax the muscles in the front of the thigh, 50 repetitions per set, 3 sets per day;  2. Hamstring Isometric Contraction Exercise: Press down with force on a cushion with the affected leg to tighten and relax the muscles in the back of the thigh, 50 repetitions per set, 3 sets per day;  3. Ankle Pump Exercise: Flex and extend the ankle joint with force, slowly and through the full range of motion, 50 repetitions per set, 3 sets per day;  4. Straight Leg Raises Training: Keep the knee extended and raise the straight leg to a point 20cm above the bed, 10 repetitions per set, hold for 5-10s each time, with a 5s rest interval, 5 sets per day; (Start with 15 raises on the first day, increase to 20 raises on the second day, 60 raises per day after the first week, and 90-120 raises per day by the end of the second week)  5. Early Weight-bearing and Adaptation Training: Starting from 1 week post-surgery, walk with the aid of crutches using a knee brace, alternating step walking, 10-20 minutes each time, 3 times a day; (Walk until feeling tired, limit relief after a 30-minute rest, postpone if there is meniscus injury until 2 weeks post-surgery)  6. Gait Balance Training: Stand on both legs for 1 minute; stand on the healthy side for 30 seconds; stand on the affected side for 30 seconds; transfer weight between both legs and front and back for 10 seconds. Repeat the cycle 4 times.  7. Heel Slide Training: Perform this training on the bed according to the range of motion of the knee joint flexion. | | |

Supplementary Table 2: Meniscal Injury Exercise Prescription for Ultimate Frisbee Enthusiasts (Months 1-6)

| **Exercise Forms** | **Aerobic Exercise** | **Core Strength Training** | **Neuromuscular Control** |
| --- | --- | --- | --- |
| **Types of Exercise** | □Long Distance Running  □Shuttle Running  □Balance Board Exercises | □Frog Jumps  □Squats  □Push-ups  □Plank Exercises | □"Single Leg Stand" Exercise  □Foot Clock Training |
| **Intensity/Frequency** | 2-3 times per week | 2-3 times per week | 2-3 times per week |
| **Time** | January to June | January to June | January to June |
| **Weekly Exercise Volume** | One Cycle per Day | One Cycle per Day | One Cycle per Day |
| **Sports Progression** | Skill Training (2-3 times per week): Skill training helps improve disc throwing and catching techniques, reducing the risk of injury. It includes the following aspects: Practice different throwing techniques, such as forehand, backhand, and sidearm throws. | Acceleration and Agility Training (2-3 times per week): Ultimate Frisbee often requires quick acceleration and agile changes of direction. Engage in drills focused on rapid acceleration and agility to enhance responsiveness and agility. | Practice of Advanced Throws such as Hammer, Scoober, and Hucks. Defensive Techniques including Marking and Intercepting. |
| **Precautions** | 1. Ultimate Frisbee Skill Training:  Throwing Skill Training: Includes different types of throwing techniques such as forehand, backhand, and overhead throws. Training should focus on accuracy, power, and technical details.  Catching Skill Training: Enhances enthusiasts' catching abilities, including high catches, low catches, and jumping catches.  Defensive Skill Training: Develops enthusiasts' defensive skills, including intercepting opponents' throws and causing turnovers.  2. Aerobic Exercise: Long Distance Running: Improves cardiovascular endurance and stamina.  3. Explosiveness and Strength Training:  Core Strength: Includes basic strength training exercises such as squats, bench presses, and deadlifts to enhance overall body strength.  Push-ups: Helps strengthen upper body and core muscles.  Frog Jumps: Used to improve explosiveness and vertical jump height.  4. Agility Training: Shuttle Running: Incorporates rapid changes of direction in training, simulating the demands of Frisbee matches  5. Flexibility and Balance Training: Balance Board Training: Improves balance and stability, reducing the risk of injury.  6. Coordinate diaphragmatic breathing to adjust breathing frequency, and perform all exercises within a pain-free range. If there is any discomfort or pain, joint locking, consult a doctor for personalized exercise prescription adjustments.  7. Start slowly, emphasizing correct form, gradually increasing to 75-85% of one-repetition maximum (1-RM). Consider high-speed (power) resistance training and lower limb functional training to increase load rates, improve speed, and strength. Use light to moderate loads (30%-70% of 1-RM). One-repetition maximum (1-RM): A standard assessment indicator of muscle strength, referring to the maximum weight or resistance lifted or resisted with proper technique for one repetition. | | |
